# How will vehicle automation and electrification affect the automotive maintenance, repair sector?

**DOI:** 10.1016/j.trip.2021.100495

**Published:** 2021-12

**Authors:** Monica Grosso, Ioan Cristinel Raileanu, Jette Krause, María Alonso Raposo, Amandine Duboz, Ada Garus, Andromachi Mourtzouchou, Biagio Ciuffo

**Affiliations:** a Joint Research Centre, European Commission, Ispra, Italy; b Independent Researcher, Milan, Italy

**Keywords:** Maintenance and repair sector, Battery electric vehicles, Autonomous vehicles, Economic impact, Road transport

## Abstract

Automation and electrification in road transport are trends that will influence several economic sectors of the European economy. The automotive maintenance and repair (M&R) sector will experience the effects of such transitions in the long term. This paper assesses the research in the road transport to derive the factors that may influence the M&R demand based on Battery Electric Vehicles (BEVs) and Autonomous Vehicles (AVs) uptake. Starting from current scientific research and grounded on interviews with experts, the paper reviews major drivers influencing M&R demand and provides indications on possible future effects. While for BEVs, previous work has been conducted to estimate the M&R cost variations, the research addressing the impacts of AVs deployment on the M&R sector is at its incipient stage, hence the views of experts were paramount to shed light on this topic. We identified a scientific consensus that BEVs have less M&R requirements compared with Conventional Vehicles (CVs). For AVs, our analysis and expert views identify some important factors influencing M&R requirements: hardware components, software that enables autonomy, the rise in vehicle kilometres travelled leading to higher wear and tear of replaceable parts, the need for adequate cleaning services, especially for fleets and shared vehicles. Further work should look at the impact of regulations and the non-insurable risks linked to M&R requirements.

## Highlights

• Electrification and automation will affect vehicles maintenance and repair (M&R).
• Drivers influencing M&R demand provides indications on possible future effects.
• Less moving parts and regenerative braking systems reduce Battery Electric vehicles’ M&R.
• Hardware and software and rise in VKT influence autonomous vehicles M&R.

## Introduction

The need to hedge the impact of vehicles on environment health and to reduce the different types of emissions will lead to great transformations in the transport sector (Alonso Raposo et al., 2019). It could continue to boost research and innovation in transport related technologies, along with the development of alternative less polluting vehicles. Among the technological innovations that the road transport sector is experiencing nowadays, electrification and automation are among the most disruptive.

Battery electric vehicles (BEVs) are seen as a good option for reducing greenhouse gas and air pollutant emissions. Still, their price remains high compared to conventional vehicles (CVs) and this makes them affordable to only a small part of consumers. Additionally, a faster fleet electrification is hindered by the limited range of BEVs and the heterogeneous distribution of charging facilities which could make longer trips challenging.

Market uptake of BEVs could be accelerated by governmental incentives deployed as observed in Santos and Rembalski (2021). Worldwide many countries have put in place subsidies schemes to encourage BEV uptake that target mainly consumers, but also producers. Such policies have mixed impacts on market development and social welfare as identified in Yang et al. (2019). Taxation policies represent additional incentives used to increase fleet electrification, including reduced rates or exemption from registration, annual ownership, fuel or other types of taxes levied on vehicles owners. The increasing number of charging facilities is deployed mostly by the private sector and in some areas through public authorities’ support (IEA, 2019).

Vehicle automation is still under development, mainly at testing stage, and needs to overcome even bigger challenges (costs, certification, litigation, liability, perception, security and privacy) (Fagnant and Kockelman, 2015) to make it acceptable and ready for full scale deployment. The costs of all the technologies needed (sensors, lidars, cameras, etc.) for an autonomous vehicle (AV) is still prohibitive (Nunes and Hernandez, 2020) and only a few companies manage to use and test their capabilities.

The technological advancements of BEVs and AVs, although at different pace, are changing and will modify even further the automotive sector with cascade effects on people’s mobility and freight transport. The possible consequences of such transitions will not remain limited to the vehicle manufacturing sector, but will go much beyond, impacting digital, energy, communication, insurance sectors to name a few (Alonso Raposo et al., 2021). Clements and Kockelman (2017) estimate the impacts on thirteen industrial sectors in the USA, showing the important economic magnitude that this disruption could create on a wider scale due essentially to accidents reduction and increase in productivity. Asselin-Miller et al. (2017) provide an indication on possible global market opportunities linked to AVs deployment indicating that major economic benefits would be experienced by Information and Communication Technology (ICT) and telecommunications sectors, as well as software industry. A study conducted in Spain by Alonso et al. (2020) on the economic impacts of AVs shows that 3 industries would experience a positive trend: freight, passenger transport and technological industry. The impacts and its knock-on effects that the technology could bring to road transport could act as potential obstacles or enablers for, not only, the transformation of the transport system but also for society (Alonso Raposo et al., 2019).

Among the sectors that could benefit or suffer from BEV and AV deployment, assessing the impacts on the maintenance and repair (M&R) sector remains challenging. A McKinsey & Company (2021) analysis indicate that fleet electrification and autonomous driving could change the importance of specific vehicles components and the frequency of maintenance and repair interventions. Still, many aspects can influence the demand in M&R services which makes it difficult to come up with a clear trend.

The structural differences between BEVs and conventional vehicles trigger modifications in the type of maintenance services needed, in terms of spare parts and accessories that require replacement during the lifespan of the electrical vehicle to ensure its adequate functioning. As presented in Dombrowski and Engel (2014) the automotive aftermarket is impacted in various ways and at multiple level (e.g., parts manufactures, distribution, workshops) by the increase in electric mobility.

While for BEVs substantial data and analysis is available, showing a possible decrease in such demand (Delucchi and Lipman, 2001, Propfe et al., 2012, Letmathe and Suares, 2017, Palmer et al., 2018), the assessment of AVs effects is even more challenging, as no full automated vehicles are deployed yet. The present paper will focus on investigating and estimating the potential impact that BEVs and AVs deployment could have on the M&R sector in Europe. Starting from current scientific research and grounded on interviews of experts in the field, the paper reviews major drivers influencing M&R service demand and provides indications on possible future effects.

After providing some background information on the M&R sector in Europe in Section 2, the methodology used in this study is presented in Section 3. Section 4 illustrates major findings of a literature review on demand and cost estimation of M&R of BEVs and AVs. In Section 5 the main results of the current analysis are presented. The key takeaways and concluding remarks are included in Section 6.

## The maintenance and repair sector in Europe^1^

In this paper, the M&R sector is defined according to the NACE Rev.2 Classification, code G4520^2^ which includes activities under the Maintenance and Repair of motor vehicles. The M&R sector represents around 0.9% of EU GDP and employs about 1,34 million persons in the EU MSs (Commission, 2018a, Commission, 2018b).

**Fig. 1:**
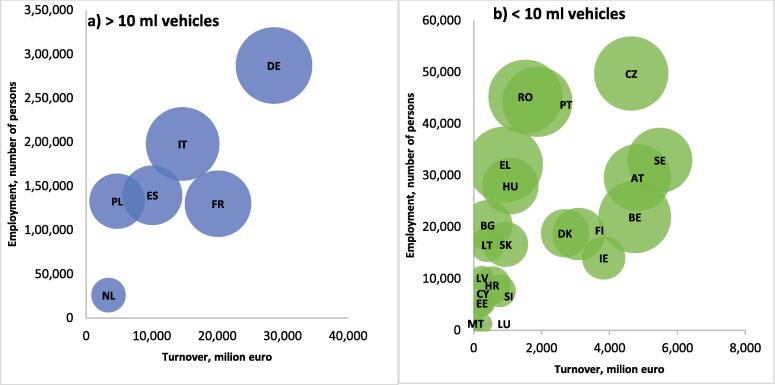
Employment and turnover of M&R sector by size of vehicle market. Chart a) refers to EU markets with stock greater than 10 million vehicles, while chart b) refers to the other EU markets. Source: Own elaboration based on data from Eurostat and JRC, reference year 2018 for employment, turnover and vehicle stock, For Finland the employment and turnover data from 2017 are used. Note: Belgium (BE), Bulgaria (BG), Czechia (CZ), Denmark (DK), Germany (DE), Estonia (EE), Ireland (IE), Greece (EL), Spain (ES), France (FR), Croatia (HR), Italy (IT), Cyprus (CY), Latvia (LV), Lithuania (LT), Luxembourg (LU), Hungary (HU), Malta (MT), Netherlands (NL), Austria (AT), Poland (PL), Portugal (PT), Romania (RO), Slovenia (SI), Slovakia (SK), Finland (FI), Sweden (SE).

The economic situation and evolution of the M&R sector should be viewed in relation to the size of the vehicle market. In this regard, the situation in the EU Member States (MSs) is diverse. Fig. 1a and 1b presents the relation between employment and turnover in the sector and the vehicle stock in the EU MSs in 2018. To ensure readability of the information, the data was separated in two figures given the high differences in the stock of vehicles. Fig. 1a presents the situation in big vehicle markets with a stock above 10 million vehicles and Fig. 1b shows medium and smaller markets. The size of the bubble represents the total number of road vehicles (cars, vans, trucks, busses, two wheelers) in 2018.

Overall, at EU 27 level the M&R sector has seen a positive evolution in the period 2011–2018. The number of M&R enterprises increased by more than 10% (with 43,300 additional companies in this period, reaching 452,830 in 2018). The growth can be attributed mainly to strong increases in Poland, France, Spain, Germany and Lithuania. Italy had the highest number of enterprises in the EU in 2018 with more than 70,400 companies, representing 15.6% of the total number of M&R enterprises in EU 27, although a small contraction was registered in the last years.

The M&R sector employs around 1.34 million persons in EU 27, showing a positive trend during the period 2011–2018, with more than 49,000 additional persons employed in the sector. EU MSs that registered strong positive employment trends in this period were Poland, Germany, Hungary and Lithuania while a reduction was observed in France, Italy, Greece and Belgium. Germany holds the highest number of people employed in the sector with more than 287,000 persons in 2018 representing 21.4% of the total EU 27 value. Additional, MSs with more than 100,000 persons employed were Italy, France, Spain and Poland. These 5 MSs represent more than 2/3 of the total number of persons employed in M&R in EU 27 and have a similar proportion in terms of the share of population to the total population living in EU 27.

The turnover of enterprises active in M&R in the EU has undergone a positive evolution between 2011 and 2018^3^^.^ The highest increase was registered by firms in Poland and Germany. The combined turnover of the top 4 states (Germany, France, Italy and Spain) represents more than 61% of the total turnover of the sector in the EU 27.

**Fig. 2:**
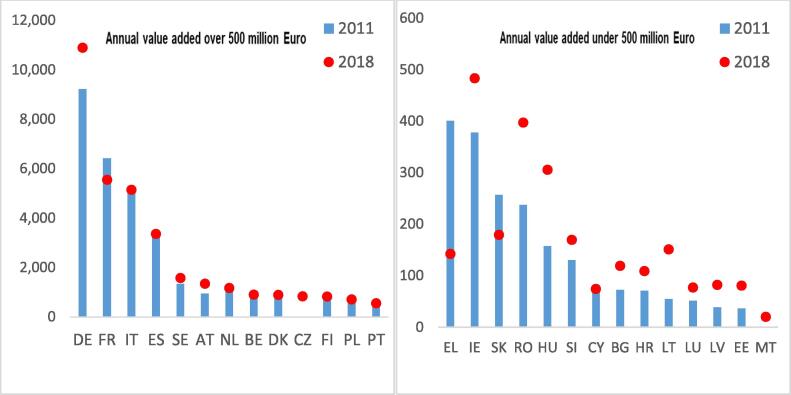
Evolution of M&R value added. Chart on the left refers to EU Member States with annual value added greater than 500 million €, while the chart on the right to the other EU MSs. Source: Own elaboration based on data from Eurostat. Finland data for 2017. Czech Republic and Malta missing data in 2011. Note: Belgium (BE), Bulgaria (BG), Czechia (CZ), Denmark (DK), Germany (DE), Estonia (EE), Ireland (IE), Greece (EL), Spain (ES), France (FR), Croatia (HR), Italy (IT), Cyprus (CY), Latvia (LV), Lithuania (LT), Luxembourg (LU), Hungary (HU), Malta (MT), Netherlands (NL), Austria (AT), Poland (PL), Portugal (PT), Romania (RO), Slovenia (SI), Slovakia (SK), Finland (FI), Sweden (SE).

In terms of value added, the situation in the M&R sector is presented in Fig. 2a and b. Germany, France, Italy and Spain hold the largest markets, with Germany leading, having more than 10,9 billion euro in value added. From 2011 to 2018, France, Greece, Belgium and Slovakia have registered the steepest decrease in terms of value added. Strong increases were registered in Germany, Austria and Sweden.

## Methodology

The analysis presented in this paper stems from an initial assessment of current scientific research in the field based on which it was possible to derive the factors that may influence the increase or decrease in M&R demand based on BEV and AV uptake. It also focuses on identifying trends and factors that can have a potential impact on M&R if AVs represent the majority of vehicles in the transport system. As BEVs and AVs differ both in terms of technology and deployment, the analysis used was different.

As the technology for BEVs is in a much-advanced phase, the analysis of previous scientific research provided sufficient evidence to sustain an estimation of M&R cost variations. In contrast, the scarcity of references addressing the impacts of AVs deployment on the M&R sector imposes a different approach and we used semi-structured interviews with automotive and transport experts to limit the literature insufficiency.

The selection of relevant literature was carried out based on the relevance to the topic and limited to the last ten years, as no major contributions could be found earlier. No geographical boundaries were defined.

**Fig. 3:**
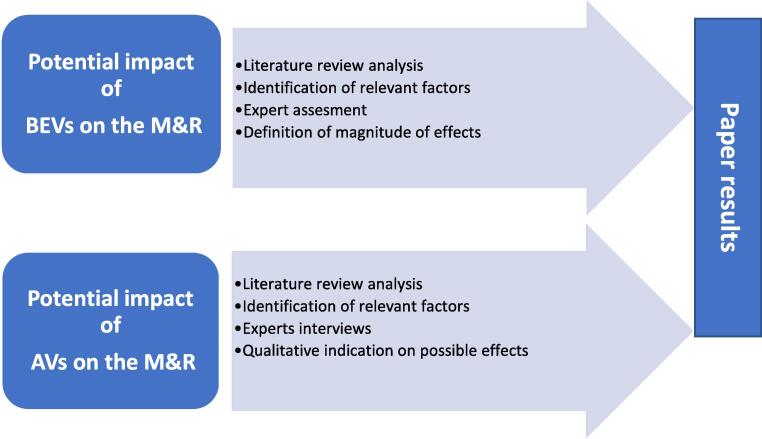
Methodological approach.

Fig. 3 illustrates the different methodological approaches used for BEVs and AVs.

In the case of BEVs, after a detailed analysis of Literature Review (LR) on the topic, the list of possible elements affecting BEVs M&R was defined and the related results assessed by 5 researchers working as transport economists at the Joint Research Centre (JRC). Through several iterative discussions, the experts convened to define an average BEVs M&R variation value.

The outcome of the AVs LR helped to develop a framework to provide a qualitative indication of the increase/decrease in the demand for M&R of AVs based on specific factors and taking into account different deployment scenarios. To test the soundness of factors, trends and changes identified in AVs literature, it was necessary to involve transport system and automotive experts that provided qualitative insights on the impact of AVs on the M&R sector.

Expert opinions collected through interviews are an important source of information and facilitate the understanding of complex issues in areas where limited knowledge is available. Such methodology has been widely applied also in transport research as documented in Zhang et al., 2021. The online interviews method was chosen to collect the views of experts that were living in various countries (e.g., Belgium, Germany, United Kingdom, South Korea). We acknowledge the limitations of the method chosen, especially in terms of the reduce richness of information produced by the interviews as described in Johnson et al. (2019), still taking into consideration the restrictions in traveling and organising face to face interviews due to COVID-19 we consider it to be the best alternative available for collecting the data. Experts in transport system and the automotive sector were identified following a purposive sampling approach as described in Etikan et al. (2016).

The interviewed participants were selected from a list of experts previously known by the authors through research events or engagement activities. In all, 30 experts were contacted by email or LinkedIn between October 2020 and January 2021, 9 experts accepted to take part in the research. Of these, 4 were from the automotive sector associations representative at EU or MS level, 3 from automotive consulting companies and 2 were working in automotive companies. All the experts interviewed had experience within the transport sector, namely in transport systems, automotive aftermarket, automotive hardware/software companies and automotive consulting. 3 of the experts had AVs related experience.

Information about the 9 experts are presented in Table 1.

**Table 1:** Details about the experts interviewed.

**Field of activity**	**Field of experience**	**Years of experience**	**Years of AV related experience**
EU Automotive Association	Automotive	20+	–
EU Automotive Association	Automotive aftermarket	15+	–
EU Automotive Association	Automotive aftermarket	20+	–
Automotive Association	Automotive	10+	–
Automotive Company	Automotive technology	5+	–
Automotive Supplier	Software automotive and AVs	5+	5+
Consulting	Automotive; AVs	20+	8+
Consulting	Automotive and consulting for automotive	5+	–
Consulting	Consulting on transport and AVs	5+	2+

Source: Own elaboration based on the information collected from the experts interviewed.

Starting from previous LR on the topic, the experts could elaborate on the factors identified and complement with additional information and data based on their experience in the transport/automotive field, moreover a section on future mobility scenarios and a concluding part for additional comments/material complemented the questionnaire (see Annex 1). These inputs have been framed in a semi-structured questionnaire that was used to guide the discussion with transport experts during the online interviews. The experts were asked to state whether a certain factor could have a positive or negative impact on the M&R sector, its ranking, from 1 (less important) to 5 (very important) and possibly to define a share of increase/decrease in cost compared to CVs (Table 2).

**Table 2:** List of papers reviewed for M&R cost of BEVs and CVs.

**Authors (year)**	**M&R costs estimations/ assumptions (% BEVs lower than CVs)**	**Country/Region**	**Type of vehicle considered in the analysis**
König et al. (2021)	28%* Small, 16%* Medium, −2%*^1^ Large and 11%* SUV	Germany	Passenger Cars
Liu et al. (2021)	At least 40%*, but increasing considering the value of vehicle	USA	Passenger Cars
Harto (2020)	50%* lifetime average	USA	Passenger Cars
Lutsey and Nicholas (2019)	57%* car, 55%* crossover, 59%* SUV	USA	Passenger Cars
Moon and Lee (2019)	50%	Korea	Passenger Cars
Van Velzen et al. (2019)	50%	The Netherlands	Passenger Cars
Palmer et al. (2018)	23% Japan; 30% California; 24% Texas; 23% United Kingdom *	Japan; California; Texas; United Kingdom	Passenger Cars
Weldon et al. (2018)	18%	Ireland	Passenger Cars and Light Commercial Vehicles
Logtenberg et al. (2018)	47%	Canada (includes regional values)	Passenger Cars
Danielis et al. (2018)	30% or 35% based on the values identified in literature	Italy	Passenger Cars
Letmathe and Suares (2017)	25–31% Small Vehicles 30–35% Medium Vehicles Class (depending on the Annual vehicle mileage)	Germany	Passenger Cars
Hoekstra et al. (2017)	70%	Netherlands	Passenger Cars
Mitropoulos et al. (2017)	30%	USA	Passenger Cars
Kleiner and Friedrich (2017)	33% (40 ton long- haulage) 46% (12 ton urban)	Germany	Trucks (40 ton and 12 ton)
Bubeck et al. (2016)	25%	Germany	Passenger Cars
Madina et al. (2016)	65%*	Spain; Germany; The Netherlands	Passenger Cars
Rusich and Danielis (2015)	50%	Italy	Passenger Cars
Gnann et al. (2014)	19% Small; 17% Medium; 16% Large and LCV*	Germany	Passenger Cars and Light Commercial Vehicles
Taefi et al. (2014)	20–30%	Germany; United Kingdom	Light Commercial Vehicles
Lebeau et al. (2013)	35%	Belgium	Passenger Cars
Macharis et al. (2013)	50%	Belgium (Brussels-Capital Region)	Light Commercial Vehicles
Davis and Figliozzi (2013)	50%*	USA	Trucks
Lee et al. (2013)	50%- 75%	USA	Trucks
Propfe et al. (2012)	19%*	Germany	Passenger cars
Egbue and Long (2012)	25%*	USA	Passenger Cars
Feng and Figliozzi (2012)	50%	USA	Commercial Vehicles - Delivery Trucks
Delucchi and Lipman (2001)	26%*	USA	Passenger cars

*The % difference was calculated based on the nominal values provided /used in the paper Source: Own elaboration based on the information collected in the BEVs Literature review; ^1^ In König et al. (2021) M&R costs reported for large BEV are higher than those for a large CV.

The experts were interviewed via internet collaboration tools between the months of November 2020 – January 2021, with the interviews lasting from 30 to 75 min. The authors put in writing the answers provided, which were then sent to each expert for approval or further adjustment. Each expert had the possibility to check his/her contribution and to clarify specific views.

## Literature review results

Previous work dealing with demand and cost estimation of M&R of BEVs and AVs has been reviewed and the main findings are presented hereafter.

### Maintenance and repair cost of battery electric vehicles

Details, calculations and specific values/percentage differences between the maintenance costs of BEVs and CVs are covered in various papers. Most of them are related to vehicle costs analysis.

Delucchi and Lipman (2001) identified M&R cost as an important part of the operational cost of a vehicle and developed a first estimation of the M&R costs for the lifecycle of a vehicle, namely 5.05 US¢/mile for a CVs (including oil but excluding inspection, cleaning and towing) and 3.72 US¢/mile for BEVs. These values represent a 26% decrease in M&R cost of BEV compared to CV.

Propfe et al. (2012) look into the Total Cost of Ownership (TCO) over a period of four years. M&R cost calculations took into account the mean time between failures /replacements and the required input for replacing specific components. The relative M&R cost of BEVs compared to CVs was estimated to be around 19% lower. In absolute values, the cost of M&R calculated for CVs was 2.892 € and for BEVs 2.348€ (over a 4-year period and with an average of 10,000 km/year driven).

More recently, Letmathe and Suares (2017) analysed the TCO for specific BEVs^4^ and CVs models grouped into small, medium, and large size vehicles. The cost for M&R was considered part of the vehicle operating expenditures along with energy consumption, other variable costs (e.g., car care), insurance, vehicle taxes, other fixed costs (e.g., renting a parking space), and the cost for battery leasing in the case of BEVs. M&R costs taken from a vehicle costs database^5^ account for: oil changes, inspections, wear and tear damages, a fixed repair annual lump sum and replacement of the starter battery^6^. The paper also considered different drivers’ profiles (occasional, normal, and frequent) based on the annual vehicle kilometres travelled. The decrease in the cost of M&R for BEVs models compared to CVs varies between 25 and 35%.

In Palmer et al. (2018), TCO and M&R costs were estimated for two countries, Japan and the UK, and two states in the USA, California and Texas. The percentage decrease of BEVs M&R costs compared with equivalent CVs is 23% in Japan and the UK, 24% in Texas and 30% in California.^7^

The table below provides a broader overview of the LR on the topic, showing the different values in M&R cost in BEVs and CVs. The BEVs TCO analyses show a general trend towards a decrease in their cost overtime, which may lead to close the cost gap with CVs in the near future (Liu et al., 2021).

### Maintenance and repair cost of autonomous vehicles

To our knowledge, previous scientific publications related to M&R demand and cost estimation associated to AVs are less abundant. Up until February 2021, there were no available papers extensively covering this topic and the potential increase/decrease comparison with CVs costs. Nonetheless hereafter we report the relevant studies identified.

Information on the M&R costs of AVs presented in Chen et al., 2016, Loeb and Kockelman, 2019 cover only the case of shared autonomous electric vehicles (SAEV). The assumption made in Chen et al. (2016) is that the M&R cost of a SAEV is the same as for a CV, namely ranging from 5.5 to 6.6 US¢/mile with a midpoint at 6.1 US¢/mile. Loeb and Kockelman (2019) look at the cost of deploying a SAEVs fleet in Austin, Texas, using similar values to Chen et al. (2016). In this paper, the vehicle cleaning cost is included in the high costs’ scenario, which accounts for 2.6 US¢/mile.

Given the incipient stage of deployment of AVs in transport networks and the challenges these vehicles must overcome to be considered safe to travel unattended the scarcity of the literature looking into the costs of AVs, and more specifically into M&R costs is understandable.

## Results of own assessment and from experts’ interviews

In this section, we illustrate the result of our analysis: we first look at BEVs and then at AVs deployment effects on the automotive M&R sector.

### Estimated impact of battery electric vehicles deployment on maintenance and repair

Overall, there is a clear consensus that M&R cost for BEVs is lower than for CVs. This cost decrease is attributed to the following factors: BEVs have fewer moving parts (Palmer et al., 2018, Logtenberg et al., 2018, Mitropoulos et al., 2017, Lebeau et al., 2013, Feng and Figliozzi, 2012), do not need oil and filters changes (Moon and Lee, 2019, Logtenberg et al., 2018, Lebeau et al., 2013) and have regenerative braking systems that have a lower impact on wear and tear of different components (Palmer et al., 2018, Logtenberg et al., 2018, Hoekstra et al., 2017, Lebeau et al., 2013).

Given the large range of values proposed in LR, from cautious ones 19% (Propfe et al., 2012), to more extreme ones 70–75% (Lee et al., 2013, Hoekstra et al., 2017) the value proposed in this paper is a cautious approach in line with the literature, which is an average value of 30% of maintenance cost reduction of BEV compared CVs.

### Estimated impact of autonomous vehicles deployment on maintenance and repair

This analysis presents an estimate of potential increase/decrease in demand for M&R for AVs. The outcome of this analysis is reported here after.

The AV deployment scenarios are the ones illustrated below, for which different degrees of effects in the M&R demand are expected.

**Autonomous conventional vehicles for private use (ACVs)** - in this scenario the automated features are installed in CVs and the majority of vehicles in the transport system continues to be owned by private individuals;

**Autonomous electric vehicles for private use (AEVs) -** in this scenario, the technology that enables the vehicle to be autonomous is installed in electric vehicles and the majority of vehicles in the transport system continues to be owned privately by individuals;

**Shared autonomous conventional vehicles (SACVs) –** in this case, CVs become autonomous and the majority of vehicles in the transport system is owned by companies/public providers and used for transport needs by individuals;

**Shared autonomous electrical vehicles (SAEVs) -** in this last scenario, AVs are electrical and the fleet is owned by companies/public providers and used for transport needs by individuals.

The actual deployment of AVs in the transport system will probably be a dynamic combination of the scenarios previously described. For the ease of the analysis, this paper estimates the impact of AVs on the M&R sector by looking at each scenario separately and disregarding the effects that could unfold when combining them.

The factors that could influence M&R demand due to AVs’ deployment have been identified in LR and are presented in Table 3.

**Table 3:** Estimation of increase /decrease in demand for M&R of AVs based on the literature reviewed.

**Factors that could influence M&R demand due to AVs deployment**	**Privately owned ACVs**	**Privately owned AEVs**	**Shared SACVs**	**Shared SAEVs**
Maintenance in general	**+/−**	**–**	**+**	**+/−**
Autonomous driving system Hardware and Software (sensors, controls, software updates and navigation, etc.)	**+**	**+**	**+**	**+**
Connectivity	**+**	**+**	**+**	**+**
Outstanding maintenance interventions (e.g. reduced probability of accidents)	**–**	**–**	**–**	**–**
Additional empty VKT (vehicle kilometres travelled)	**+**	**+**	**+**	**+**
Cleaning	**N.C.**	**N.C.**	**+**	**+**
Reduced fleet size	**–**	**–**	**+**	**+**
Increase demand for travel and the total VKT	**+**	**+**	**+**	**+**
Lower acceleration and deceleration	**–**	**–**	**–**	**–**

+ (increase); **−** (decrease); +/**−** (difficult to establish); N.C. (no change).

Source: Own elaboration based on the information collected in the AVs Literature review.

Overall demand for maintenance could increase in the SACVs scenario, considering the anticipated increase in vehicles kilometres travelled (VKT) for fleets and ride sharing services as documented in Henao and Marshall (2019) and the induced wear and tear of components. For the SAEV and ACV scenarios, the changes in demand for maintenance are difficult to establish. For the AEV scenario, overall M&R demand could decrease in line with the estimations of reduced M&R costs for EVs compared to CVs.

New maintenance service niches could be developed to check directly and remotely the functioning of the autonomous driving systems, to update various software and to ensure permanent connectivity to different networks and environment elements. All these services could increase M&R demand and costs in all scenarios, in line with the analysis in Litman (2020). To alleviate the burden of such expenses, these services could be provided in assistance packages that would imply regular payments.

Previous work on AVs’ benefits and threats (Litman, 2020, Gkartzonikas and Gkritza, 2019, Wadud et al., 2016) highlights the reduction in the number of crashes and probability of accidents, this would imply a decreasing need for outstanding M&R interventions in all scenarios considered.

Narayanan et al. (2020) summarizes the estimations regarding the expected variations in VKT in the case of shared autonomous vehicles and identifies fifteen studies that anticipate an increase of VKT which ranges from + 2% (Kondor et al., 2019) up to + 89% (International Transport Forum, 2015) and five studies that predicted a decrease in VKT up to a maximum of −45% (Lokhandwala and Cai, 2018).

The empty travel of AVs could be considered as an influencing factor. For shared fleets, this could be due to relocation, pick-up drives, or return-to-base drives. While in case of private use, the vehicles could be travelling empty searching for a parking place or avoiding to pay high parking fees (Bösch et al., 2018). Empty VKT are likely to increase the demand for M&R for AVs in all scenarios.

The cleaning cost has also been considered as an additional expense associated to shared AVs deployment. The cost of such services will increase the M&R demand in shared scenarios as regular cleaning would be necessary to ensure an adequate level of comfort for users, while cleaning is unlikely to impact additionally the privately owned AVs (Litman, 2020).

Autonomous taxis and shared autonomous mobility services have the potential to substitute privately owned and used vehicles and to lead to a decrease in fleet size as anticipated in Boesch et al., 2016, Burghout et al., 2015. This has the potential to reduce the demand for M&R from private users, but could increase the demand in the shared scenarios. Wadud et al. (2016) indicate that an increase in travel demand could occur due to the reduction in costs of driver’s time and rise in the number of people that could be served by AVs. This could increase the demand for maintenance in all scenarios considered.

Previous literature (Wadud, 2017) indicates that AVs could be programmed to accelerate and decelerate smoothly imposing less pressure on wear/tear of specific components; this factor could further reduce the demand for M&R in all scenarios.

Overall, the two electric AVs scenarios, either privately owned or shared fleets, could lead to a reduction of M&R costs. This could essentially be attributed to the electrical components, rather than to the automation technology. The privately owned electric AVs would have a slightly lower M&R costs than shared electric AVs when including the cost of cleaning.

In order to validate the LR findings and to gain further insights on the factors, trends and changes identified for M&R of AVs, we organised semi-structured interviews with transport system and automotive experts.

The experts were firstly asked about their general opinion on whether AVs would have higher or lower M&R requirements compared to CVs and what could be the elements/reasons for such a variation. The answers showed that it remains highly difficult to estimate accurately the impact of AVs’ deployment on M&R. Most experts indicated that the expected decrease in accidents would lead to less M&R interventions, but repairing/replacing and calibration/recalibration of complex hardware components installed in AVs could prove to be costly procedures.

Next, based on LR findings, the respondents were asked to provide their view on a selected list of factors that have the potential to influence the demand for M&R of AVs. The results obtained are presented in Table 4, where for each factor, the overall tendency of increase or decrease in M&R demand is presented, together with a ranking of the different factors, where 1 indicates that the factor is not important at all and 5 that the factor is very important.

**Table 4:** Summary of experts’ estimations on the impact of AVs on M&R.

**Factors**	**Impact of AVs on M&R demand compared to CVs**	**Ranking** (1 not important at all, 5 very important)	
**AVs Hardware**	**+**	**4**	
**AVs Software**	**+**	**4**	
**Connectivity**	**/**	**2**	
**Additional empty VKT**	**/**	**1**	
**Increased demand in VKT**	**+**	**3**	
**Cleaning services**	**+**	**4**	
**Others**	Fleet electrification	**–**	
	Cybersecurity needs can add to the M&R costs of AVs.	**+**	
	M&R of AVs will require new special equipment and people with appropriate skills to use it	**+.**	

+ (increase); **−** (decrease); +/**−** (difficult to establish); NC (no change); / no significant impact foreseen.

Source: Own elaboration based on the information provided by experts interviewed.

The experts were also asked to estimate the possible share of cost variation of the identified factors compared to CVs. Still, the high degree of uncertainty associated with AV deployment and the lack of available data on this last point impeded the respondents to provide reliable information. The overview of the retrieved information is summarised in Table 4.

For each of the factors, the additional insight provided by the experts is presented below. The factors listed as “Others” were identified spontaneously by experts during the interviews.

#### Hardware for autonomous driving

Hardware components and software programs that will enable vehicles to be fully autonomous are seen as highly influencing factors in the future that could determine an increase in M&R demand.

The AVs will be equipped with more various hardware components (sensors for camera, radar, lidar, etc.) than the average CV on the road today and many of its parts require highly accurate calibration to ensure proper functioning that could lead to an increase in M&R demand.

Experts with a background in automotive companies and the aftermarket sector pointed out that hardware wear and tear could have less influence on M&R of AVs, while the hardware calibration/recalibration could constitute an important factor. This view is based on the experience with Advanced Driver Assistance Systems (ADAS) available nowadays^8^. ADAS hardware calibration represents a post-repair or replacement service process which ensures the proper functioning of vehicle sensors and which is done static, dynamic or in combination.

Automotive consultancy experts share the view of a potential increase in M&R of AVs determined by hardware components, mainly attributed to the high number of components and their complexity. Higher number of components increases system complexity and hence adds possible operational issues, leading to a raise of replacing and calibrating costs.

The views of the experts interviewed are in line with the findings of the LR which emphasized the importance of hardware components for providing a vehicle with autonomous capabilities (Koopman and Wagner, 2017, Pendleton et al., 2017).

#### Software for autonomous driving

The software part of AV is also regarded as an important factor for M&R by all the experts interviewed. Most of them anticipate that processes like software Over the Air updates (OTA)^9^ will represent a common feature and could have an impact on the M&R demand.

Panel of experts working in automotive companies have emphasized that OTA would change the nature of M&R services and determine the implementation of new processes. This could bring regulatory challenges that have a potential impact on M&R costs, particularly in the introductory phase.

Use of OTA has multiple implications for the M&R sector, since vehicles could no longer be required to have regular physical checks of specific software applications. For vehicle owners, OTA reduces some indirect costs related to maintenance such as time and money spent for service centre visits. Still, particular concerns about OTA are related to security and protection of the software from external threats and about the need for rigorous testing and regulatory validation of system modifications.

Specialized literature contains a series of proposals to overcome the OTA security concerns such as: special secure protocol for OTA in Nilsson and Larson (2008), blockchain technology in Baza et al. (2019), STRIDE^10^ which is a scheme for OTA software updates over cloud that is specifically designed for AVs in Ghosal et al. (2020).

Additionally, some experts pointed out that AVs could be equipped with systems and software enabling remote diagnostics and failure detection. Predictive diagnostics as described in Hackner and Lehle (2017) increase availability and optimize the maintenance intervals of vehicles, transforming unplanned breakdowns into predictable maintenance interventions which further have the potential to reduce maintenance time and costs.

However, automotive consulting experts indicated that a stronger development of software applications in the short and medium term is needed to ensure that AVs overcome the challenges of adequately operating in extraordinary weather and traffic conditions.

#### Connectivity

The impact of connectivity technologies on the M&R of AVs is still difficult to assess and the current deployment situation provides limited information. Regular check-ups of connectivity features would be deemed necessary, but the actual costs and relevance for M&R interventions cannot be currently estimated.

Connectivity could enhance vehicle automation (Ha et al., 2020) and would be an embedded part in the deployed AVs. Connectivity for AVs could include the remote vehicle ignition, doors unlocking, fuel use, and data exchange among vehicles (V2V), to the infrastructure (V2I), to the cloud (V2C) and to everything (V2X).

Most of the experts interviewed indicated that connectivity would be embedded in the AVs, but this factor will have a small influence on the M&R. One expert suggested that connectivity does not represent a distinct factor and that it could be split into the hardware and software parts.

#### Additional empty vehicles kilometres travelled

Additional empty travel is seen as a less important factor for the M&R of AVs by the experts interviewed. Most experts suggested that the expected impact may be further reduced through optimization of AVs operation and allocation.

Deployment of AVs, either private or shared, in the transport system may lead to additional empty travel for various reasons such as: repositioning, use of AVs as mobility service robots, return to origin if parking is not available at the destination (Meyer et al., 2017). Empty travel can partially offset some of the benefits of AVs and may increase congestion (Liu et al., 2017).

The views expressed by the experts are in line with the research efforts to develop optimization-based strategies that decrease empty kilometres travelled and traveller waiting time in the case of shared-use AVs mobility service (Hyland and Mahmassani, 2018).

#### Increased demand in vehicles kilometres travelled

All the experts in the panel consider the estimated increase in VKT as a relevant factor for the M&R of AVs. A rise in VKT could lead to a higher usage of replaceable parts in AVs and an increase in terms of M&R demand. One expert indicated that, for autonomous EVs to cope with the estimated increase in VKT, battery management would play a very important role.

Deployment of AVs may increase the demand for VKT based on many determinants. Harb et al. (2021) present a comprehensive review of the AVs literature, including their impact on travel behaviour and VKT, and, although various methodologies are used in the papers analysed, a large majority indicated an increase of the VKT with a range from 1% to 90%. Taiebat et al. (2019) provide examples of main determinants for an increase in VKT, based on literature: induced travel demand, new demand from underserved travellers, a response to reduced cost of driving, empty travelling, etc.

#### Cleaning services

The experts viewed cleaning as an important factor that will increase the operational cost of AVs. Importance of the cleaning could be intensified by sanitary concerns over the spread of viruses and diseases (e.g. Covid-19 pandemic). The hygienic concerns represent an important challenge, especially for shared AVs and could impact the trust and use of shared services if regular and suitable cleaning procedures are not deployed.

Although cleaning is not at the core of M&R activities, the cleaning services may be a relevant factor in the case of AVs. Litman (2020) sees cleaning as an important factor that would determine a change in materials used for the interior of vehicles and as an operational cost, especially for autonomous taxis that will require more frequent cleaning. One expert mentioned that cleaning could be relevant for ensuring the adequate functioning of various sensors the AVs are equipped with.

#### Other additional factors

During the interviews, the experts had the possibility to complement the list of identified factors with additional elements that could come from their background or expertise.

The additional factors mentioned were:

*Changes in technology and delivery of services*: M&R for AVs could require new special equipment for performing specific tasks (e.g. sensor calibration) or could imply the use of automated inspection systems; some maintenance services (upgrades/updates) could be performed remotely.

*Cybersecurity needs and concerns*: considering the important roles played by software and connectivity in the functioning of AVs, an increase in cybersecurity needs and threats is foreseen. These may lead to additional costs for reducing or removing any negative effects which consequently could have a positive effect on the demand for M&R.

*Transformation of the workforce in the M&R sector*: deployment of AVs may transform the nature of the M&R services and could increase the need to upskill and regularly train the sector’s employees in order to keep up with the technological changes. The investment in human capital may impact the costs of M&R services for AVs in the short and medium term.

*Fleet electrification*: large scale use of electric AVs could lead to a decrease in M&R costs, primarily attributed to the fact that electric vehicles have lower M&R needs compared with conventional ones. This aspect was highlighted spontaneously by some of the interviewed experts, without knowing that this topic was also within the scope of this research, and reconfirmed the findings of the BEVs analysis.

### Expert views on possible scenarios for the deployment of automated vehicles and additional relevant aspects

Another aspect investigated with the experts interviewed for this paper was the suggested deployment scenarios of AVs. The experts were presented with the four possible scenarios identified in the LR stage, namely autonomous conventional vehicles for private use (ACVs), autonomous electric vehicles for private use (AEVs), shared autonomous conventional vehicles (SACVs) and shared autonomous electrical vehicles (SAEVs).

About half of the experts indicated that SAEVs scenario could be the one for a mature market of AVs, still, the cost of the shared service will play an important role. The remaining experts pointed out that AEVs for private use could reach around 70–80% of the global vehicle market, especially based on personal attributes and preferences of individuals. Some mentioned that the autonomous capabilities could be installed on conventional vehicles, but this would represent a reduced part of the vehicle stock.

The experts’ views indicated that the transition to electrical vehicles is seen as inevitable and this would bring changes and challenges also in terms of demand and cost for M&R.

Additional relevant aspects regarding the future deployment of AVs and its anticipated impact on M&R include:

• the high importance of the regulatory framework of the AVs ecosystem, especially regarding new M&R services suppliers, future market players, software providers, shared services providers, etc., which needs to empower and protect consumers;
• the idea that AVs could be designed and built for the specific purpose the vehicle would serve (e.g. shared AVs could have a different design than privately used ones) and this usage profile could further impact the wear and tear of different components;
• the fact that deployment of AVs would require and determine massive shifts and changes at industry level. This could include new or different business choices such as general maintenance centres linked or run by the original equipment manufacturers vs. the specialization of M&R service providers on particular task/components.

## Conclusions

In 2018, the automotive M&R sector employed more than 1.34 million people in the EU 27 MS and this labour force provided services for a stock of vehicles close to 308 million. Although BEVs represented less than 1% of that stock, the push for electrification and cleaner vehicles in the future would have a transformative impact on the M&R sector among others. AVs are still in a testing phase, but their future deployment could bring further changes in the M&R sector too.

In this paper, we analysed the scientific literature to identify potential factors and trends that may influence M&R demand based on BEV and AV uptake. BEVs and AVs remain very different in technological and deployment terms; these differences influenced the approach used in assessing their impacts on the M&R sector. For BEVs, literature review and experts’ judgment supported the definition of the relevant factors and the quantification of their order of magnitude. For AVs, after identifying relevant influencing factors through LR, experts’ interviews were used to validate LR findings and to gather additional insights.

We identified a scientific consensus that BEVs have less M&R requirements compared with CVs, since BEVs have fewer moving parts, do not need oil and filter changes and have regenerative braking systems that reduce the wear and tear of different components. In terms of magnitude, a realistic assumption is that the M&R cost for BEVs is at least 30% lower than that of CVs.

For AVs, our analysis indicates the following important factors influencing M&R requirements: hardware components, software that enables a vehicle to drive autonomously, the rise in VKT that may lead to a higher wear and tear of replaceable parts, and the need for adequate cleaning services, especially for fleets and shared vehicles. Additional factors are the connectivity and the empty vehicles kilometres travelled.

In terms of possible deployment scenarios of AVs, the interviews with transport and automotive experts have indicated that the future is electric; moreover, it could be distinguished by shared mobility options or private ones, or by a combination of the two. Also, experts pointed out that ACVs could represent a minor part of the vehicle stock in the future.

The LR and experts’ inputs highlighted some additional relevant aspects that could shape the M&R of AVs, more specifically: regulatory aspects; cybersecurity challenges; the transformation of the workforce and skills requirements of future employees in the sector; and the impact of system diagnostic features and of preventive/predictive maintenance.

Further work should look at the impact of regulations and the non-insurable risks linked to M&R requirements as these factors were not covered by the current analysis and were not discussed during the interviews with the experts since they had a more technical background. Such factors are important for M&R of BEVs, and would be particularly relevant for AVs considering the expected shift and increase in liability costs as presented in Shannon et al. (2021) and in responsibility – based allocation of liability costs according to Pütz et al. (2018).

Another area that needs further investigation and evidences is linked with the potential structural transformation of the M&R sector with the deployment of AVs. Specifically, the emergence of fewer, bigger and specialised providers that have easier access to data and information collected by the vehicles sensors could be detrimental to the evolution and growth of the small and medium-size enterprises active in the sector. While sufficient evidence exists to define effects that BEVs will bring into the automotive M&R sector, the incipient stage of full vehicle automation deployment impedes a clear understanding the impact that such technology will bring into the M&R sector. As piloting and testing activities proceed towards full automation, further research should be carried out to deepen the knowledge and produce estimates on the overall impact that such technological disruption will cause in the M&R sector.

## CRediT authorship contribution statement

**Monica Grosso:** Conceptualization, Data curation, Formal analysis, Investigation, Methodology, Supervision, Writing – original draft, Writing – review & editing. **Ioan Cristinel Raileanu:** Conceptualization, Data curation, Formal analysis, Investigation, Methodology, Visualization, Writing – original draft, Writing – review & editing. **Jette Krause:** Conceptualization, Investigation, Writing – review & editing. **María Alonso Raposo:** Writing – review & editing. **Amandine Duboz:** Writing – review & editing. **Ada Garus:** Writing – review & editing. **Andromachi Mourtzouchou:** Writing – review & editing. **Biagio Ciuffo:** Funding acquisition, Project administration, Resources, Validation, Writing – review & editing.

## Declaration of Competing Interest

The authors declare that they have no known competing financial interests or personal relationships that could have appeared to influence the work reported in this paper.

## Footnotes

1 The analysis in this section is limited to EU-27 MS since 1 February 2020. It does not include information related to the situation of the maintenance and repair sector in the United Kingdom.

2 NACE REV. 2 Statistical classification of economic activities in the European Community, Available at: https://ec.europa.eu/eurostat/documents/3859598/5902521/KS-RA-07–015-EN.PDF

3 Exception made for Belgium, Greece and France.

4 With at least 100 new vehicles registration in the German market.

5 ADAC (2015). More details about this database are available at: https://www.adac.de/rund-ums-fahrzeug/autokatalog/

6 For cars used more than 30,000 km/year.

7 Calculations based on the nominal values provided in the paper.

8 ADAS Sensor Calibration Increases Repair Costs available at: https://www.aaa.com/autorepair/articles/adas-sensor-calibration-increases-repair-costs

9 OTA represents remote enhancements brought to cars firmware and software that provide access to new features or fix issues and security gaps.

10 STRIDE represents a secure and scalable software update technique developed and proposed in Ghosal et al., (2020)
